# Response to Yang and Riva-Cambrin

**DOI:** 10.1097/PR9.0000000000000876

**Published:** 2021-01-13

**Authors:** Esther M. Pogatzki-Zahn, Maryam Yahiaoui-Doktor, Winfried Meissner, Peter K. Zahn, Alexander Schnabel

**Affiliations:** a University Hospital of Muenster, Muenster, Germany; b Maryam Yahiaoui-Doktor: Institute for Medical Informatics, Statistics and Epidemiology, University of Leipzig, Leipzig, Germany; c Department of Anaesthesiology and Intensive Care, Jena University Hospital, Jena, Germany; d Department of Anaesthesiology and Intensive Care Medicine, Palliative Care Medicine and Pain Management, Berufsgenossenschaftliches Universitätsklinikum Bergmannsheil GmbH Bochum, Ruhr University Bochum, Germany

We thank the colleagues Yang and Riva-Cambrin for their comments^11^ related to our recently published database analysis.^9^ The main aim of our article^9^ was to identify risk factors and (if possible) to develop a clinically easy to use risk score relevant for acute pain after surgery. We used the largest multicenter international PAIN OUT database for this research question^13^ because we aimed to identify risk factors not limited to one specific surgery nor to one institution in a specific country. Furthermore, because the clinical relevance of pain intensity as the most important patient-reported outcome measure (PROM) in postoperative pain has been criticized,^6,8,10^ we also examined other, possibly more important, PROMs such as “time spent in severe pain” or “wished to have received more pain treatment”; these PROMs are assessed in PAIN OUT by using the validated IPO questionnaire that has been translated into many different languages.^7,13^ Logistic binary regression and exploratory factor analysis enabled us to develop a very easy to use prediction model with a decent (but still not perfect) predictive value, which was thereafter validated in a second cohort.

Of course, besides the clear advantages (like the extremely high number of data sets available, very strict and therefore comparable data assessment, multicenter approach, multicultural approach, and clinical relevant PROMs beyond pain intensity), such an approach still has a number of limitations. We therefore definitively agree to some of the criticisms by Yang and Riva-Cambrin that we discussed in detail and would like to refer to our article where we clearly refer to these limitations. First, we discussed^9^ that data collection was (only) performed on the first day after surgery (including the psychological predictors that are criticized in the letter). No doubt, we need to verify the relevance of these factors (and/or irrelevance related to preoperative psychological assessments) in the future.

Second, it was criticized that the external validity of the prediction model was limited because “country” was a relevant predictor such that the prediction model can only be used in those countries participating in the PAIN OUT project. We in fact think that this is a strength—PAIN OUT is an international project and data from 26 countries were included.^13^ All risk factors we included in our score turned out to be risk factors irrespective of the country and the additional risk of the country itself gives additional information at the end. As discussed,^9^ the country was indeed the most important risk factor highlighting the influence of cultural aspects, such as different pain relief and treatment expectations in different countries. Thus, we would instead suggest to validate other scores, which were limited to data from just one country or even just one center^3–5,12^ before generalization and recommending a “world-wide” use.

Third, we thank the colleagues Yang and Riva-Cambrin^11^ for mentioning the TRIPOD guidelines.^2^ We are currently trying to increase the predictability of our score in a prospective multicenter international trial; there we will certainly adjust our approach to this useful guideline.

The development of scores aiming to predict poor acute (or chronic) pain outcome after surgery is rare. Most scores derived earlier are hampered by aspects such as the small number of patients assessed,^1^ mix of preoperative and postoperative variables to calculate the risk^1^ making it impossible to assess the risk already before surgery, limiting the risk assessment to one specific surgical procedure alone,^4,5,12^ or developing the score in one country (or even one hospital) alone.^1,3–5,12^ We tried to overcome all of these limitations with—of course—accepting some others. Thus, we do not believe that our risk score is the final answer in the development of a model for the prediction of patients with poor postoperative pain outcome, but it is an important step on that way.

Our main aim for the future is—based on our data here^9^ and those by others—to increase the predictive value of our score, for instance, by incorporating one or 2 more factors without losing the ease of use, the suitability in many different countries, and the applicability for almost all surgical procedures. The goal is a general score for all surgical procedures with good-to-excellent predictability and applicability in many countries. This is a slightly different approach to that performed by Yang et al. for one surgical procedure.^12^ Yes, we appreciate that as a neurosurgeon, it is important to use such a surgical-specific score for a shared-decision approach related to the surgical treatment. By contrast (or maybe better complementary to this), we would like to use it for a shared-decision approach related to the perioperative pain management modifiable by anesthetists. Our vision would therefore not be to work “against each other” or an “either - or,” but rather a “building on each other” approach between surgeons and anesthetists to assess the patient's risk and—within the framework of a “shared-decision approach”—prevent and treat the patient complementary.

## Disclosures

The authors have no conflicts of interest to declare.

## References

[1] Althaus A Hunrichs-Rocker A Chapman R Arranz Becker O Legering R Simanski C eber F Moser KH Joppich R Trojan S Gutzeit N Neugebauer E. Development of a risk index for the prediction of chronic post-surgical pain. Eur J Pain 2012;16:901–10. 22337572 10.1002/j.1532-2149.2011.00090.x

[2] Collins GS Reitsma JB Altman DG Moons KGM. Transparent reporting of a multivariable prediction model for individual prognosis or diagnosis (TRIPOD): the TRIPOD statement. BJC 2015;112:251–9. 25562432 10.1038/bjc.2014.639 PMC4454817

[3] Kalkman CJ Visser K Moen J Bonsel GJ Grobbee DE Moons KG. Preoperative prediction of severe postoperative pain. PAIN 2003;105:415–23. 14527702 10.1016/S0304-3959(03)00252-5

[4] Montes A Roca G Sabate S Lao JI Navarro A Cantillo J Canet J. Genetic and clinical factors associated with chronic postsurgical pain after hernia repair, hysterectomy, and thoracotomy: a two-year multicenter cohort study. Anesthesiology 2015;122:1123–41. 25985024 10.1097/ALN.0000000000000611

[5] Pan PH Tonidandel AM Aschenbrenner CA Houle TT Harris LC Eisenach JC. Predicting acute pain after cesarean delivery using three simple questions. Anesthesiology 2013;118:1170–9. 23485992 10.1097/ALN.0b013e31828e156f PMC3951732

[6] Pogatzki-Zahn EM Schnabel K Kaiser U. Patient-reported outcome measures for acute and chronic pain: current knowledge and future directions. Curr Opin Anaesthesiol 2019;32:616–22. 31415046 10.1097/ACO.0000000000000780

[7] Rothaug J Zaslansky R Schwenkglenks M Komann M Allvin R Backstrom R Brill S Buchholz I Engel C Fletcher D Fodor L Funk P Gerbershagen HJ Gordon DB Konrad C Kopf A Leykin Y Pogatzki-Zahn E Puig M Rawal N Taylor RS Ullrich K Volk T Yahiaoui-Doktor M Meissner W. Patients' perception of postoperative pain management: validation of the International Pain Outcomes (IPO) questionnaire. J Pain 2013;14:1361–70. 24021577 10.1016/j.jpain.2013.05.016

[8] Scher C Meador L Van Cleave JH Carrington Reis M. Moving beyond pain as the fifth vital sign and patient satisfaction scores to improve pain care in the 21^st^ century. Pain Manag Nurs 2018;19:125–9. 29249620 10.1016/j.pmn.2017.10.010 PMC5878703

[9] Schnabel A Yahiaoui-Doktor M Meissner M Zahn PK Pogatzki-Zahn EM. Predicting poor postoperative acute pain outcome in adults: an international, multicentre database analysis of risk factors in 50005 patients. PAIN Rep 2020;5:e831. 32766467 10.1097/PR9.0000000000000831 PMC7390596

[10] Wilkerson RG Kim HK Windsor TA Mareiniss DP. The opioid epidemic in the United States. Emerg Med Clin North Am 2016;34:e1–e23. 27133253 10.1016/j.emc.2015.11.002

[11] Yang MMH Riva-Cambrin J. Prediction tools for postoperative pain. PAIN Rep 2021;6:e875. 33490846 10.1097/PR9.0000000000000875 PMC7810507

[12] Yang MMH Riva-Cambrin J Cunningham J Jette N Tolulope TS Soroceanu A Lewkonia P Jacobs WB Casha S. Development and validation of clinical prediction score for poor postoperative pain control following elective spine surgery. J Neurosurg Spine 2020. doi: 10.3171/2020.5.SPINE20347 [Epub ahead of print]. 32932227

[13] Zaslansky R Rothaug J Chapman CR Backstrom R Brill S Fletcher D Fodor L Gordon DB Komann M Konrad C Leykin Y Pogatski-Zahn E Puig MM Rawal N Ullrich K Volk T Meissner W. PAIN OUT. The making of an international acute pain registry. Eur J Pain 2015;19:490–502. 25132607 10.1002/ejp.571

